# Functional status mediates the association between peripheral neuropathy and health-related quality of life in individuals with diabetes

**DOI:** 10.1007/s00592-017-1077-8

**Published:** 2017-11-28

**Authors:** Tessa Riandini, Hwee Lin Wee, Eric Y. H. Khoo, Bee Choo Tai, Wilson Wang, Gerald C. H. Koh, E. Shyong Tai, Subramaniam Tavintharan, Kurumbian Chandran, Siew Wai Hwang, Kavita Venkataraman

**Affiliations:** 1 Saw Swee Hock School of Public Health, National University of Singapore, Tahir Foundation Building, 12 Science Drive 2, Singapore, 117549 Singapore; 2 Department of Pharmacy, Faculty of Science, National University of Singapore, 18 Science Drive 4, Singapore, 117559 Singapore; 3 Department of Medicine, Yong Loo Lin School of Medicine, National University of Singapore, 1E Kent Ridge Road, NUHS Tower Block, Level 10, Singapore, 119228 Singapore; 4 Department of Orthopaedic Surgery, Yong Loo Lin School of Medicine, National University of Singapore, and University Orthopaedics Hand & Reconstructive Microsurgery Cluster, NUHS Tower Block, Level 11, 1E Kent Ridge Road, Singapore, 119288 Singapore; 5 Diabetes Centre, Khoo Teck Puat Hospital, 90 Yishun Central, Singapore, 768828 Singapore; 6 Department of Medicine, Ng Teng Fong General Hospital, 1 Jurong East Street 21, Singapore, 609606 Singapore; 7 SingHealth Polyclinics - Bukit Merah, 163 Bukit Merah Central, Singapore, 150163 Singapore

**Keywords:** Diabetic polyneuropathy, Functional status, Health-related quality of life, Muscle strength, Postural balance, Range of motion, Type 2 diabetes mellitus

## Abstract

**Aims:**

To examine differences in health-related quality of life (HRQoL) between patients with and without diabetic peripheral neuropathy (DPN), and whether these differences can be explained by functional deficits.

**Methods:**

This was a cross-sectional study of 160 patients with type 2 diabetes mellitus, 80 with DPN and 80 without. Assessments included HRQoL (health utility score derived from EQ-5D-5L), functional status measurements [muscle strength, timed up and go (TUG), five times sit-to-stand (FTSTS), functional reach, body sway velocity] and self-reported balance confidence [Activities-specific Balance Confidence (ABC) scale].

**Results:**

Mean utility scores were 0.67 ± 0.14 and 0.77 ± 0.16 in patients with and without DPN, respectively (*p* < 0.001). Patients with DPN had lower great toe extensor strength (6.4 ± 1.8 vs 7.6 ± 2.8 lbs, *p* = 0.001), greater body sway velocity (2.40 ± 1.31 vs 1.90 ± 0.52 mm/s, *p* = 0.002), slower TUG (12.1 ± 4.6 vs 10.1 ± 2.3 s, *p* < 0.001) and FTSTS (15.8 ± 5.8 vs 13.9 ± 5.4 s, *p* = 0.03) scores, and lower ABC score (73.4 ± 21.3 vs 82.6 ± 16.9, *p* = 0.003), compared to those without DPN. On stepwise multiple regression, DPN status, FTSTS, body sway velocity, BMI, diabetes duration, pain, and gender explained 38% of HRQoL variance. Addition of ABC score into the model explained 45% of variance. Results from structural equation modelling showed that DPN had direct effects on HRQoL and indirect effects through FTSTS, body sway velocity, and ABC score, with *χ* ^2^ = 8.075 (*p* = 0.044), root mean square error of approximation = 0.103 (lower bound 0.015, upper bound 0.191), Comparative Fit Index = 0.966, Tucker–Lewis Index = 0.887, and Standardized Root Mean Square Residual = 0.053.

**Conclusions:**

Patients with DPN have worse HRQoL compared to patients without DPN, partly mediated by functional status parameters. Effective interventions targeting functional status may be beneficial in improving HRQoL in these patients.

**Electronic supplementary material:**

The online version of this article (10.1007/s00592-017-1077-8) contains supplementary material, which is available to authorized users.

## Introduction

Diabetes has been associated with reduced quality of life [1], with further reductions as the disease progresses and complications start to occur [2]. One common complication in individuals with diabetes is diabetic peripheral neuropathy (DPN), affecting up to 50% individuals with diabetes [3]. The most common type of peripheral neuropathy is distal symmetric sensorimotor polyneuropathy, which usually affects the distal extremities first before extending proximally, causing sensory loss in a glove and stocking pattern [4]. DPN patients are at high risk of foot infections and ulcerations [4] as well as falls and injuries, which may in turn lead to amputation and death [5].

Individuals with DPN are known to have poorer health-related quality of life (HRQoL) [6, 7]. We, and others, have also previously shown that DPN, including early-stage DPN, can reduce quality of life even in the absence of pain [6, 7]. Individuals with DPN are recognized to have sensorimotor deficits, including reduced proprioceptive sense [8], ankle mobility, range of motion [9, 10], and muscle strength [11], leading to reduced balance [12], gait and mobility alterations [9, 13], and increased risk of falls [14]. It is unclear, however, if these functional deficits in individuals with DPN are associated with, and responsible for, reduction in HRQoL.

In this study, therefore, we aim to quantify the reduction in HRQoL associated with DPN, identify functional deficits among patients with DPN in terms of muscle strength, range of motion, balance functional tasks, and balance confidence, and examine the role of these functional deficits in the reduction of HRQoL. We hypothesized that patients with DPN have functional deficits compared to patients with diabetes but without DPN, and these deficits account for the reduction of HRQoL.

## Methods

### Study population

This was a cross-sectional study of 160 participants with known type 2 diabetes, of whom 80 had DPN and 80 did not. Participants were recruited from those attending specialist outpatient clinics in 4 tertiary care hospitals and from 1 polyclinic in Singapore between July 2014 and April 2017. Ethical approval was obtained from the National Healthcare Group Domain Specific Review Board and SingHealth Centralised Institutional Review Board. Written informed consent was obtained from all participants prior to study initiation.

Study inclusion criteria were age between 40 and 79 years and physician-diagnosed type 2 diabetes mellitus. Participants were excluded if they had one of the following: foot ulceration/infection/amputation; any medical contraindication for physical activity and/or physiotherapy; non-diabetic neuropathy; non-diabetes and non-neuropathy-related orthopaedic, surgical, or medical conditions affecting functional mobility and balance; severe diabetes complications including advanced retinopathy; and end-stage renal disease requiring dialysis. DPN was defined as documentation of two or more insensate sites out of 10 points on both feet using 10-g Semmes–Weinstein monofilament testing and/or vibration perception threshold of 25 Volts or more on neurothesiometer testing, with or without neuropathy symptoms [15].

### Data collection

All participants completed questionnaires on socio-demographic characteristics (age, gender, ethnicity, education, employment, marital status, monthly household income, housing type), medical history (hypertension, dyslipidemia, heart disease, peripheral arterial disease, arthritis), The International Physical Activity Questionnaire (IPAQ) short form for assessment of physical activity, Michigan Neuropathy Screening Instrument (MNSI) for assessment of DPN symptoms, and EQ-5D-5L. Participants also underwent physical examination that included measurement of height, weight, basic vital signs, foot examination, and assessment of physical function.

### EQ-5D-5L

The EQ-5D-5L is a generic measure of HRQoL, comprising a health descriptive component and a visual analogue scale. The health descriptive component consists of five dimensions, one each on mobility, self-care, usual activities, pain/discomfort, and anxiety/depression. Each item has five levels of response: no problem, slight problem, moderate problem, severe problem, and extreme problem [16]. The questionnaire used was a self-administered English version validated for Singapore [16]. All subjects were conversant in English. EQ-5D scores can be summarized into a single index value, the health utility score, using population preference weights. This index ranges between 0 and 1, where 1 represents perfect health and 0 represents death. Since there is no value set available yet for Singapore, the value set for Japan was used to calculate health utility scores in our study [17].

### Physical function assessment

Muscle strength at ankle and big toe were measured for dorsiflexion at the joint using a handheld dynamometer (micro FET3, Hoggan Scientific, Utah, USA) positioned on the dorsum of the foot and proximal phalanx of the great toe, respectively, with the participant seated and the knee extended. Range of motion at ankle was measured for dorsiflexion–plantar flexion at the joint using a handheld inclinometer (MicroFET3, Hoggan Scientific, Utah, USA) placed on the dorsum of the foot, with the participant seated, the knee extended and starting with the ankle fully plantar flexed. Range of motion at knee was measured for flexion–extension at the joint using the handheld inclinometer placed on the lower third of the back of the leg of interest, with the participant standing with the knee extended at the start. After a mock run, two trials were conducted for each measure and the mean taken.

Participants were assessed on their functional capability using the timed up and go (TUG) [18], five times sit-to-stand (FTSTS) [19], and functional reach [20] tests. The TUG is a test of mobility and measures the time taken by a participant to rise from a seated position, walk three metres forward, walk back and be seated. The FTSTS is a test of functional strength and measures the time taken for a participant to rise from and return to a seated position five times in a row. The functional reach test is a measure of balance during tasks and measures the distance a person can reach forward with his arm while standing without losing balance. A practice test was administered before the actual test for each measure.

A balance platform (Accugait, AMTI, USA) was used to measure average body sway velocity with the participant standing with eyes closed for 2 min. Two runs were conducted for each participant and the mean taken.

Balance confidence was measured using the Activities-specific Balance Confidence (ABC) scale [21], a 16-item instrument that assesses individuals’ confidence in performing daily or routine activities without losing their balance. Each item is rated from 0 to 100% in terms of level of confidence, and the total score is the average of all individual item scores.

### Statistical analysis

Sample size estimation based on regression with anticipated effect size for health utility score using 0.15 and 10 predictors was *n* = 118. Independent sample *t* tests were used to compare the central tendency of continuous variables between DPN groups, and *χ* ^2^ tests were used to compare proportions. Differences in functional status between DPN and non-DPN groups were presented as mean difference (MD) with the corresponding 95% CI. EQ-5D results were presented both as proportions of participants reporting problems by DPN status and as health utility score. Bivariate associations with the health utility score were examined using simple linear regression. Stepwise multiple linear regressions were used with health utility score as the dependent variable, and DPN status, functional status and demographic or health-related variables with significant bivariate associations with health utility score (*p* < 0.1) as independent variables, to identify key predictors of health utility score. History of peripheral arterial disease and arthritis were not considered due to their small proportions in the study population. Two models were constructed with differing functional status parameters included: in Model 1, only directly measured parameters of functional status with significant associations with health utility score (*p* < 0.1) were included; and in Model 2, a self-report measure of balance confidence (the ABC score) was included in addition to directly measured functional status. To examine inter-relationships between DPN status, functional measures, and HRQoL, we constructed a model depicting hypothesized relationships between these parameters, using only the variables found significant in the multivariable linear regression, excluding confounding variables. This predefined model was tested using structural equation modelling (SEM) with maximum likelihood estimation method. Based on variables in model 2, DPN status was entered as an observed exogeneous variable, whereas HRQoL, FTSTS, body sway velocity, and ABC score were entered as observed endogeneous variables. Goodness-of-fit of the final model was assessed using likelihood ratio of model versus saturated (*χ* ^2^) test, root mean square error of approximation (RMSEA), Comparative Fit Index (CFI), Tucker–Lewis Index (TLI), and standardized root mean squared residual (SRMR) statistics. All statistical analyses were done using STATA/IC 14.0 (StataCorp LLC, USA).

## Results

There were 353 potentially eligible participants in the study, of which 176 were willing to be screened, 9 were not eligible, and 7 dropped out after screening. In total, 160 participants, 80 with DPN and 80 without DPN, were included in the study and analysed. Participant characteristics are presented in Table 1. Participants in the DPN group were significantly older with mean age of 64 ± 6 years compared to participants in the non-DPN group (60 ± 7 years). Majority of participants were male, Asian Indian, with secondary school education and above, currently married, with history of hypertension and dyslipidemia. Age, ethnicity, and employment status differed significantly between DPN and non-DPN groups (*p* = 0.002, 0.0043, and 0.003, respectively), while other variables did not. Mean diabetes duration was higher in the DPN group (*p* < 0.001). Based on responses to the MNSI history, DPN and non-DPN groups significantly differed in the proportions who experienced numbness (DPN: 74%, non-DPN: 30%), burning pain (DPN: 46%, non-DPN: 20%), pain while walking (DPN: 45%, non-DPN: 25%), prickling sensation (DPN: 25%, non-DPN: 11%), and weakness (DPN: 56%, non-DPN: 21%). Additionally, DPN participants reported symptoms of sensitivity to touch (23%) and muscle cramps (75%), although these did not significantly differ from the non-DPN group.

**Table 1:** Socio-demographic characteristics of participants

Variables	DPN (*n* = 80)	Non-DPN (*n* = 80)	*p* value^a^
Mean age, years (SD)	64.01 (6.23)	60.63 (7.23)	0.002*
Gender, *n* (%)			
Female	34 (42.50)	33 (41.25)	0.873
Male	46 (57.50)	47 (58.75)	
Ethnicity, *n* (%)			
Indian	66 (82.50)	55 (68.75)	0.043*
Others	14 (17.50)	25 (31.25)	
Mean diabetes duration, years (SD)	16.43 (10.81)	10.31 (9.21)	< 0.001*
Mean HbA1c, % (SD), mmol/mol (SD)^b^	8.62 (1.86), 71 (20.3)	8.40 (1.55), 68 (16.9)	0.417
Mean LDL cholesterol, mmol/L (SD)^c^	2.49 (0.90)	2.52 (0.84)	0.873
Mean HDL cholesterol, mmol/L (SD)^d^	1.16 (0.30)	1.13 (0.27)	0.649
Mean triglyceride, mmol/L (SD)^d^	1.69 (0.85)	1.73 (0.81)	0.872
Mean total cholesterol/HDL cholesterol ratio^d^	3.97 (1.15)	4.02 (0.92)	0.821
Mean BMI, kg/m^2^ (SD)	28.08 (5.35)	28.79 (5.43)	0.408
Mean MNSI examination score (SD)	1.57 (0.69)	0.01 (0.11)	≤ 0.001*
Mean MNSI history score (SD)	2.95 (1.54)	1.63 (1.60)	< 0.001*
Mean overall physical activity, MET-min/week (SD)	2347.31 (1632.69)	2479.43 (1818.32)	0.629
Experienced fall(s) in the previous 4 weeks, *n* (%)	3 (3.75)	1 (1.25)	0.311
Ever smoker, *n* (%)	15 (18.75)	11 (13.75)	0.391
Ever drinker, *n* (%)	11 (13.75)	12 (15.00)	0.822
Secondary school education and above, *n* (%)	51 (63.75)	55 (68.75)	0.504
Currently married, *n* (%)	53 (66.25)	59 (73.75)	0.121
Employed, *n* (%)	29 (36.25)	48 (60.00)	0.003*
Monthly household income > 2000 SGD, *n* (%)^e^	22 (27.50)	26 (32.50)	0.131
Housing categories, *n* (%)			
1–2 rooms	19 (23.75)	9 (11.25)	0.219
3 rooms	16 (20.00)	20 (25.00)	
4 rooms	22 (27.50)	24 (30.00)	
5 rooms or above	23 (28.75)	27 (33.75)	
Self-reported history of comorbidities, *n* (%)			
High blood pressure	55 (68.75)	50 (62.50)	0.405
High cholesterol/lipids	55 (68.75)	52 (65.00)	0.614
Heart disease	21 (26.25)	16 (20.00)	0.348
Peripheral arterial disease	7 (8.75)	2 (2.50)	0.086
Arthritis	7 (8.75)	12 (15.00)	0.222

^a^ Calculated using independent *t* test for continuous variables and ^*χ*2^ test for categorical variables

^b^ *n* = 159

^c^ *n* = 69

^d^ *n* = 70

^e^ 18 participants (11.25%) refused to answer

**Fig. 1:**
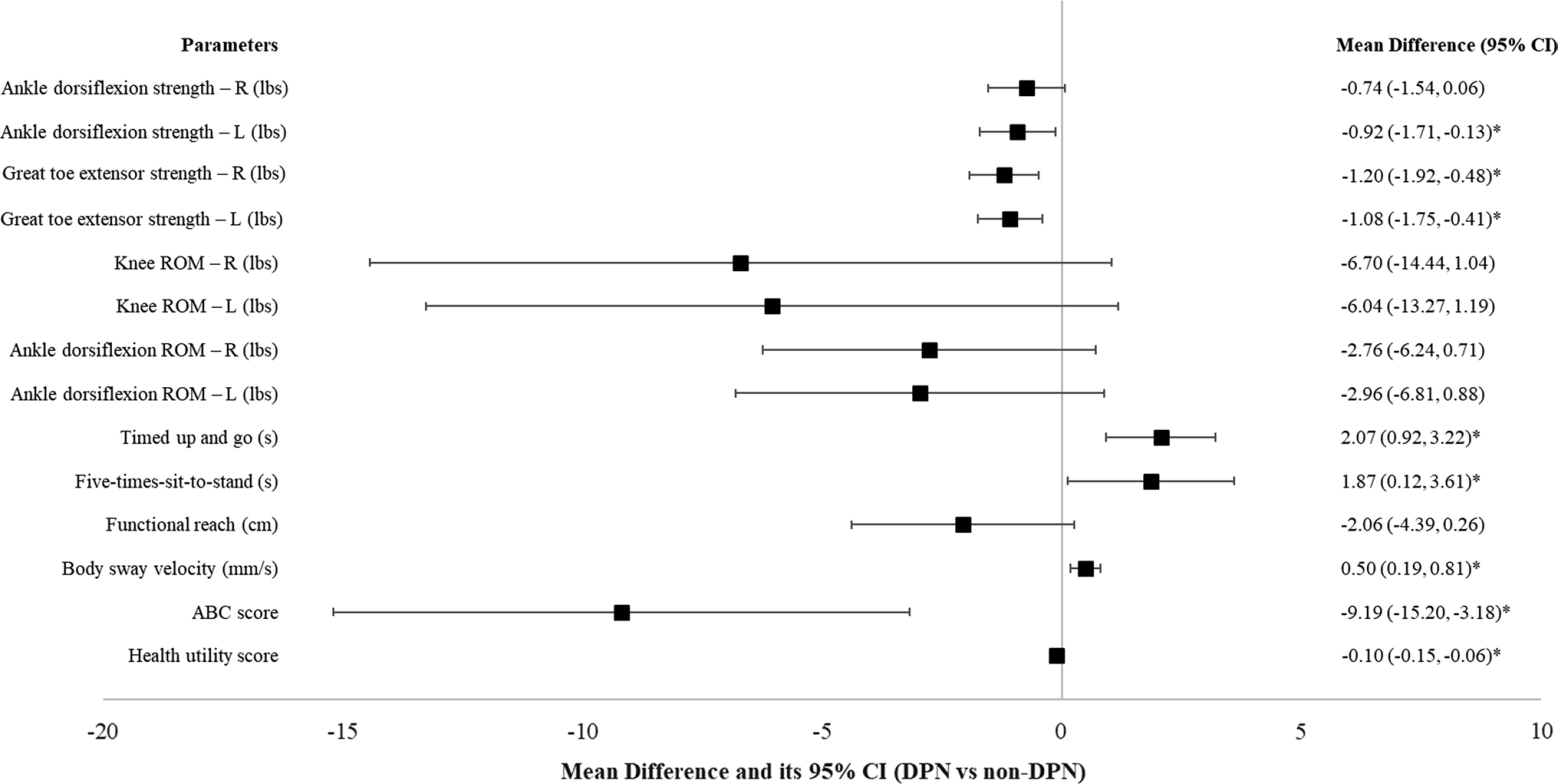
Functional status and HRQoL differences between DPN and non-DPN groups. ***R*** **right**, ***L*** **left** . * ***p*** **<** **0.05**

Participants with DPN had lower ankle dorsiflexion strength (right − 0.74 lbs, 95% CI − 1.54, 0.06 lbs, *p* = 0.068; left − 0.92 lbs, 95% CI − 1.71, − 0.13 lbs, *p* = 0.023) and great toe extensor strength (right − 1.2 lbs, 95% CI − 1.92, − 0.48 lbs, *p* = 0.001; left − 1.08 lbs, 95% CI − 1.75, − 0.41 lbs, *p* = 0.002), poorer performance on TUG test (2.07 s, 95% CI 0.92, 3.22 s, *p* < 0.001) and FTSTS test (1.87 s, 95% CI 0.12, 3.61 s, *p* = 0.036), as well as greater body sway velocity while standing with eyes closed (0.50 mm/s, 95% CI 0.19, 0.81 mm/s, *p* = 0.002) (Fig. 1, Supplementary Table 1). They also had significantly lower health utility score (− 0.10 points, 95% CI − 0.15, − 0.06 s, *p* < 0.001) compared to participants without DPN. HRQoL differed in DPN and non-DPN groups in all dimensions except pain/discomfort (Supplementary Table 1).

Unadjusted estimates of EQ-5D predictors are listed in Supplementary Table 2. In the multivariable analysis, only DPN status, FTSTS, body sway velocity, BMI, gender, diabetes duration, and burning pain were significant predictors of health utility score with explanation of 37.80% of total variance in HRQoL in Model 1 (Table 2). Addition of ABC score in Model 2 resulted in explanation of 45.35% of total variance in HRQoL (Table 2). In this model, FTSTS turned out to be non-significant (*p* = 0.254), probably due to stronger correlation between ABC score and health utility score as well as strong correlation between ABC score and FTSTS of − 0.544 (*p* < 0.001). Correlations between ABC score and functional measures ranged from 0.0456 < |*r*| < 0.5440. Age of participants, although significantly different between DPN and non-DPN groups, was not significantly associated with HRQoL (Supplementary Table 2). Employment status was significantly associated with HRQoL on bivariate analysis, but did not appear as a significant predictor in both models. Diabetes duration significantly differed between the two groups and was associated with HRQoL with borderline significance, after adjusting for functional status and other confounding factors. Burning pain was no longer significant in Model 2.

**Table 2:** Independent factors associated with HRQoL

Model	Variables	Estimate^a^	95% CI		*p*	Adj. *R* ^2^
Model 1^b^	DPN status	0.055	0.012, 0.099	0.012		0.3780
	Sit–stand 5 times	− 0.006	− 0.010, − 0.003	0.001		
	Body sway velocity	− 0.028	− 0.048, − 0.008	0.007		
	BMI	− 0.004	− 0.008, − 0.001	0.020		
	Gender	− 0.079	− 0.121, − 0.037	< 0.001		
	Diabetes duration	− 0.002	− 0.004, − 0.000	0.049		
	Burning pain	− 0.053	− 0.098, − 0.008	0.022		
Model 2^c^	DPN status	0.046	0.006, 0.087	0.025		0.4535
	Sit–stand 5 times	− 0.002	− 0.006, 0.002	0.254		
	Body sway velocity	− 0.021	− 0.040, − 0.002	0.029		
	ABC score	0.003	0.002, 0.004	< 0.001		
	BMI	− 0.004	− 0.007, − 0.000	0.032		
	Gender	− 0.064	− 0.104, − 0.025	0.002		
	Diabetes duration	− 0.002	− 0.004, 0.000	0.069		
	Burning pain	− 0.036	− 0.079, 0.007	0.101		

^a^ Beta coefficient from multivariable linear regression

^b^ Variables considered: DPN status, ankle dorsiflexion strength, knee ROM, TUG, FTSTS, body sway velocity, age, BMI, gender, diabetes duration, ethnicity, smoking status, drinking status, education level, working status, income level, history of hypertension, burning pain

^c^ Variables considered: DPN status, ankle dorsiflexion strength, knee ROM, TUG, FTSTS, body sway velocity, ABC score, age, BMI, gender, diabetes duration, ethnicity, smoking status, drinking status, education level, working status, income level, history of hypertension, burning pain

**Fig. 2:**
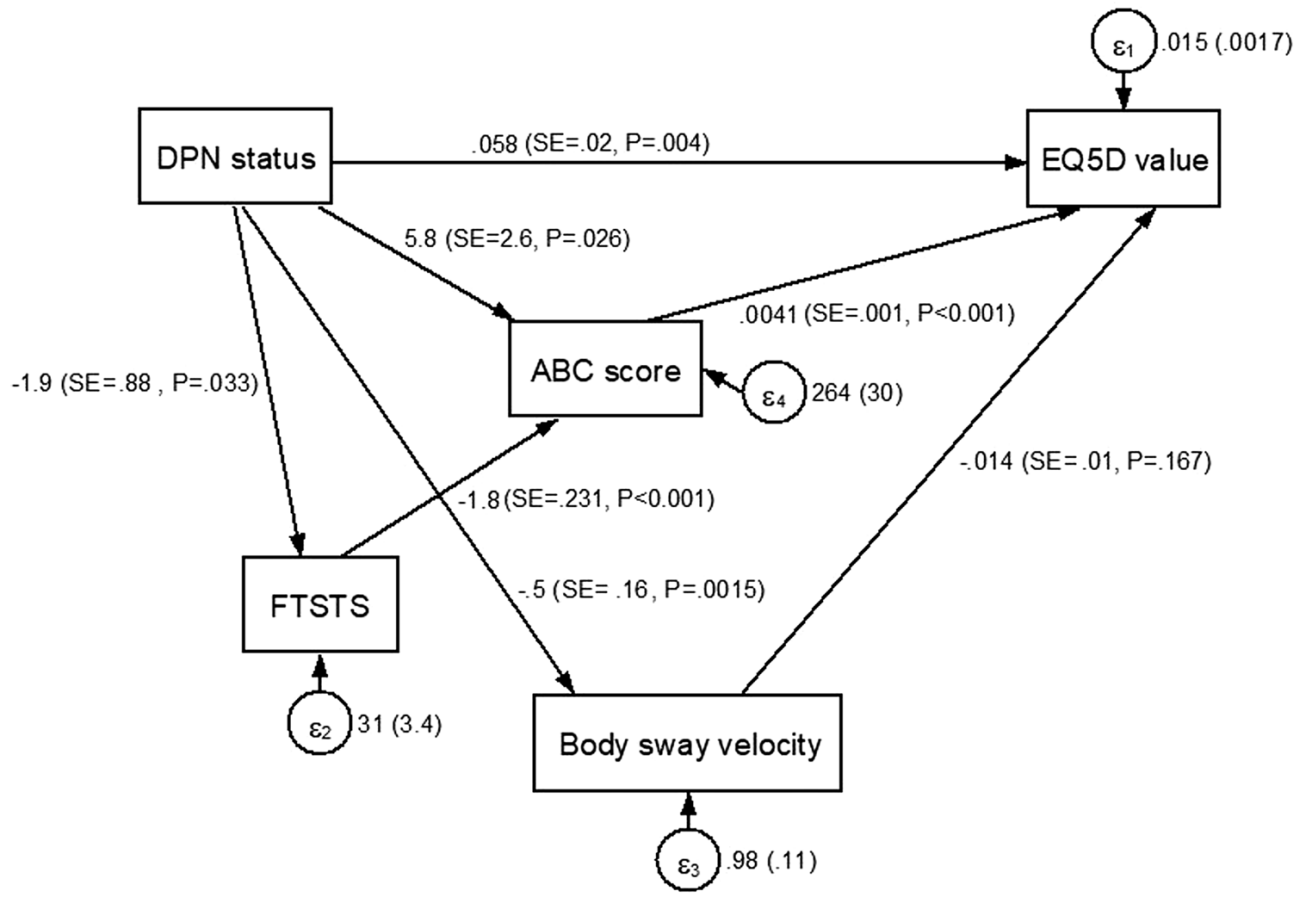
SEM analysis of HRQoL predictors. Path diagram showing inter-relationships between DPN status, functional status, and HRQoL. Boxes represent observed variables, single-headed arrows represent hypothesized causal relationships, and circles represent error terms/residuals. Reported values for each path: effect estimate (SE, *p* value); reported values for error terms (*ε*_1_ − *ε*_4_): raw variance (SE)

Structural equation modelling of the final model showed that DPN status had direct association with HRQoL, as well as indirect associations through functional status. There were significant individual paths from DPN status to FTSTS (*β* = − 1.9, SE = − 0.17, *p* = 0.033), from FTSTS to ABC score (*β* = − 1.8, SE = − 0.52, *p* < 0.001), and from ABC score to HRQoL (*β* = 0.0041, SE = 0.52, *p* < 0.001), suggesting that FTSTS and ABC score mediated the relationship between DPN and HRQoL (Fig. 2). All other paths were significant and consistent in terms of the magnitude and direction of associations with the multiple linear regression model, except for the paths from body sway velocity to HRQoL which did not reach significance (*β* = − 0.014, SE = − 0.091, *p* = 0.17) (Supplementary Table 4). The overall model showed reasonably good fit with *χ* ^2^ = 8.075 (*p* = 0.044), RMSEA = 0.103 (lower bound 0.015, upper bound 0.191), CFI = 0.966, TLI = 0.887, and SRMR = 0.053.

We confirmed the associations between functional status and HRQoL by repeating the regression analysis in the DPN group only (Supplementary Tables 5 and 6). The directions of associations and effect sizes were similar to that observed for the entire group; however, not all associations were significant in this subgroup due to the much smaller sample size. Similar to what was observed in the model for the entire group, the effect of sit–stand five times was attenuated when ABC score was put into the model.

## Discussion

This study demonstrated that individuals with DPN had a significantly lower HRQoL and lower functional status compared to those having diabetes without DPN. Balance confidence, FTSTS, and body sway velocity were the functional measures significantly associated with HRQoL, and these measures explained a portion of the association between DPN and HRQoL.

Mean utility score in the non-DPN group in this study was similar to that reported in Europe among people with diabetes patients without complications (mean = 0.74, SD = 0.27) [22]. The utility scores among patients with DPN in this study, on the other hand, were higher than the mean utility score of 0.5 ± 0.3 reported by Gore et al. [23], among patients with DPN. A possible reason for this difference is that the participants in that study comprised a wider range of DPN severity levels with patients included as long as they could reliably fill up the questionnaire, whereas participants in our study were limited to those without ulceration, amputation, or other overt sequelae of DPN. The utility scores in the DPN group in this study, however, were comparable to the scores reported in patients with other conditions from this region, such as breast cancer (mean = 0.78, SD = 0.16) [24], age-related macular degeneration (mean = 0.89, SD = 0.14) [25], and end-stage renal disease (mean = 0.60, SD = 0.21) [26]. It is evident, therefore, that the impact of DPN on HRQoL could be similar or even worse than other serious conditions.

In our sample, domains of mobility, self-care, usual activity as well as anxiety were associated with DPN, while pain was not. We, and other researchers, have previously shown that DPN is associated with significant reduction in HRQoL [27], with greatest reductions seen in the physical function/mobility [6, 7] and pain [28] domains. In individuals with mild or asymptomatic DPN, we have previously demonstrated that physical function is the quality of life domain most affected [6]. Our findings are therefore consistent with previous work in this area.

We found that functional measures, such as FTSTS and body sway velocity, were associated with the reduction in HRQoL and partly mediated the association between DPN status and HRQoL. It is well recognized that DPN leads to specific sensorimotor deficits [29], with consequent limitations in balance or postural stability [12], functional strength [30], gait and mobility [9]. However, few studies have empirically demonstrated the link between either specific neurological deficits or functional measures with HRQoL in individuals with DPN. To our knowledge, only one previous pilot study has reported on this relationship, finding significant associations between HRQoL (assessed by EQ-5D-3L) and functional mobility (assessed by chair sit–stand, timed up and go, and 6-m walk tests) in older patients with diabetes, though this was not the primary study aim [31]. While the role of functional status in HRQoL has been previously demonstrated in patient populations with known functional debility, like the elderly [32] and those with multiple sclerosis [33], ours is one of the first studies to formally investigate the role of functional status in patients with DPN on HRQoL and examine the inter-relationships between DPN, functional status, and HRQoL. It is worth noting, however, that the true casual relationships among these variables could be complicated and reciprocal. For example, it is possible that lower HRQoL in patients with diabetes may alter their self-care and diabetes management behaviours, which in turn could put them at greater risk of developing complications and further decrease HRQoL.

Balance confidence was a key functional capacity measure that was strongly and independently associated with HRQoL. As a psychological indicator of a person’s self-efficacy in undertaking activities that involve maintaining balance, balance confidence is known to correlate with functional status [34], ambulatory behaviours [35], and fall risk [36]. In our analysis, balance confidence was significantly correlated with functional status measures and dominated the relationship with HRQoL as the strongest predictor. Based on our results, the mediating paths from FTSTS to ABC score and from ABC score to HRQoL were both significant. This might explain why FTSTS became non-significant when ABC score was included and suggests mediation between FTSTS and HRQoL by balance confidence. ABC score, however, did not mediate the relationship between body sway velocity and HRQoL; therefore, balance confidence appears to be a mediator of certain functional measures only.

Socio-economic factors like employment status, income, and educational status showed significant associations with HRQoL on bivariate analysis, but not on multivariable analysis. Similar associations have been reported previously in patients with diabetes [37, 38] and underline the need for tailored clinico-social interventions in specific subgroups to improve health outcomes. However, since most studies have been cross-sectional in nature, it is also unclear whether employment and income levels are the cause or effect of DPN and HRQoL status.

The significantly poorer HRQoL in patients with DPN, and the important role of balance confidence in HRQoL reductions, strongly suggests the need for functional and balance confidence interventions to improve HRQoL. Balance and endurance training have clearly been demonstrated to be beneficial for motor and sensory symptoms in DPN [39]. Exercise, and especially tai chi, has also been found to be beneficial to improve balance confidence in older adults [40]. However, other exercise training such as aerobic, resistance, and endurance training without a specific balance component do not seem to have effects on HRQoL, at least in patients with diabetes [41]. It would be interesting to examine whether targeted balance and endurance training can impact balance confidence and HRQoL in patients with DPN. If found effective, this would be a very useful adjunct therapy to offer patients with DPN, for whom there is currently no specific management available except pain relief. The potentially mediating role of balance confidence to some functional measures such as FTSTS, however, would necessitate intervention programs to achieve improvement in functional status of a magnitude sufficient to increase balance confidence before it can subsequently improve HRQoL.

This study has several strengths. We examined a comprehensive set of objective and patient-reported functional status measures in patients with DPN to examine their contribution to HRQoL. The inclusion and exclusion criteria applied in this study allowed accurate classification of DPN and exclusion of comorbidities that might significantly confound the association between DPN and HRQoL. However, certain limitations must also be acknowledged. This was a cross-sectional study, and no causal inferences can be drawn about the associations reported, including the relationship between DPN status and functional deficits. DPN status was defined using 10-g Semmes–Weinstein monofilament and neurothesiometer testing; thus, we could have missed identifying patients with early, milder DPN as well as patients with predominantly small fibre neuropathy, or other atypical diabetic neuropathies which would not be detected using these definitions. The results, therefore, may not be generalizable to all DPN populations. Also, there was no further quantification of the severity of DPN using other methods such as nerve conduction study or needle electromyography. However, the definition used here is the same as in our previous work on diabetes complications and HRQoL, where we demonstrated that DPN was associated with the greatest reductions in HRQoL, even in the absence of pain. We did not assess neuropathic pain in detail, though pain and other symptoms were assessed through the MNSI. The sample also had disproportionate representation of specific gender and ethnic groups, which may limit the generalizability of the findings. However, this is unlikely to reduce the internal validity of our observations. Analysis with structural equation modelling might be underpowered leading to inconsistency of body sway velocity results between linear regression and structural equation modelling. However, we were unable to assess the sample size required for the final model to reach consistent results for all components.

In conclusion, this study has demonstrated that individuals with DPN have significantly lower HRQoL, which is partially explained by reduction in balance confidence and increase in body sway velocity. Based on our findings, interventions that increase functional status and balance confidence may prove useful to improve HRQoL in individuals with DPN. As reduction in HRQoL contributes greatly to the burden of the disease, interventions to improve HRQoL should be aggressively investigated.

## Electronic supplementary material

Below is the link to the electronic supplementary material.

Supplementary material 1 (DOCX 42 kb)

## Abbreviations

DPN: Diabetic peripheral neuropathy
HRQoL: Health-related quality of life
MNSI: Michigan neuropathy screening instrument
IPAQ: International Physical Activity Questionnaire
TUG: Timed up and go
FTSTS: Five times sit-to-stand
ABC: Activities-specific balance confidence
MD: Mean difference
SEM: Structural equation modelling
RMSEA: Root mean square error of approximation
CFI: Comparative Fit Index
TLI: Tucker–Lewis Index
