# Supplementary material for: Functional status mediates the association between peripheral neuropathy and health-related quality of life in individuals with diabetes
Source: Acta Diabetol. 2017 Nov 28;55(2):155–64. doi: 10.1007/s00592-017-1077-8 (PMC5816102; doi:10.1007/s00592-017-1077-8)
Supplement: Supplementary file 1 — Supplementary material 1 (DOCX 42 kb) [file 592_2017_1077_MOESM1_ESM.docx]

**Supplementary Table 1. Functional status and HRQoL of participants**

| **Variables** | **DPN**  **(n=80)** | **Non-DPN**  **(n=80)** | **MD (95% CI)^a^** | **p-value^b^** |
| --- | --- | --- | --- | --- |
| Mean strength, lbs (SD) |  |  |  |  |
| Ankle dorsiflexion – right | 9.47 (2.46) | 10.21 (2.66) | -0.74 (-1.54, 0.06) | 0.068 |
| Ankle dorsiflexion – left | 8.99 (2.35) | 9.91 (2.70) | -0.92 (-1.71, -0.13) | 0.023* |
| Great toe extensor – right | 6.43 (1.75) | 7.63 (2.76) | -1.20 (-1.92, -0.48) | 0.001* |
| Great toe extensor – left | 6.13 (1.87) | 7.22 (2.40) | -1.08 (-1.75, -0.41) | 0.002* |
| Mean range of motion, deg (SD) |  |  |  |  |
| Knee – right | 102.34 (19.84) | 109.04 (28.85) | -6.70 (-14.44, 1.04) | 0.089 |
| Knee – left | 103.14 (18.58) | 109.18 (26.92) | -6.04 (-13.27, 1.19) | 0.101 |
| Ankle – right | 78.90 (10.61) | 81.66 (11.63) | -2.76 (-6.24, 0.71) | 0.119 |
| Ankle – left | 78.55 (13.40) | 81.51 (11.16) | -2.96 (-6.81, 0.88) | 0.131 |
| Mean timed up and go, seconds (SD) | 12.13 (4.63) | 10.06 (2.35) | 2.07 (0.92, 3.22) | <0.001* |
| Mean five-times-sit-to-stand, seconds (SD) | 15.75 (5.76) | 13.89 (5.41) | 1.87 (0.12, 3.61) | 0.036* |
| Mean functional reach, cm (SD) | 23.5 (6.98) | 25.56 (7.87) | -2.06 (-4.39, 0.26) | 0.082 |
| Mean body sway velocity, mm/s (SD) | 2.40 (1.31) | 1.90 (0.52) | 0.50 (0.19, 0.81) | 0.002* |
| Mean Activity-specific Balance Confidence score (SD) | 73.42 (21.33) | 82.61 (16.90) | -9.19 (-15.20, -3.18) | 0.003* |
| Mean health utility score (SD) | 0.67 (0.14) | 0.77 (0.16) | -0.10 (-0.15, -0.06) | <0.001* |
| EQ5D dimensions (%)^c^ |  |  |  |  |
| Mobility |  |  |  |  |
| No problems | 30.00 | 58.75 | - | 0.001* |
| Some problems | 58.75 | 38.75 | - |  |
| Severe problems | 11.25 | 2.50 | - |  |
| Self-care |  |  |  |  |
| No problems | 75.00 | 93.75 | - | 0.004* |
| Some problems | 22.50 | 5.00 | - |  |
| Severe problems | 2.50 | 1.25 | - |  |
| Usual activity |  |  |  |  |
| No problems | 58.75 | 77.50 | - | 0.010* |
| Some problems | 40.00 | 18.75 | - |  |
| Severe problems | 1.25 | 3.75 | - |  |
| Pain/discomfort |  |  |  |  |
| No problems | 17.50 | 31.25 | - | 0.122 |
| Some problems | 68.75 | 58.75 | - |  |
| Severe problems | 13.75 | 10.00 | - |  |
| Anxiety/depression |  |  |  |  |
| No problems | 35.00 | 60.00 | - | 0.003* |
| Some problems | 48.75 | 35.00 | - |  |
| Severe problems | 16.25 | 5.00 | - |  |

^a^MD: Mean Difference

^b^Calculated using independent t-test for continuous variables and chi-square test for categorical variables

^c^EQ5D-5L was summarized into 3 levels: 1-No problems; 2,3-Some problems; 4,5-Severe problems

**Supplementary Table 2. Bivariate associations with EQ5D utility scores***

| **Variables** | **Estimate** | **95% CI** | **p-value** | **R^2^** |
| --- | --- | --- | --- | --- |
| DPN status | 0.103 | 0.056, 0.149 | <0.001 | 0.1081 |
| Ankle dorsiflexion strength - right (lbs) | 0.011 | 0.001, 0.020 | 0.028 | 0.0300 |
| Great toe extensor strength - right (lbs) | 0.009 | -0.001, 0.019 | 0.093 | 0.0177 |
| Ankle range of motion - right (deg) | 0.001 | -0.001, 0.003 | 0.461 | 0.0034 |
| Knee range of motion - right (deg) | 0.002 | 0.001, 0.003 | 0.002 | 0.0612 |
| Ankle dorsiflexion strength - left (lbs) | 0.011 | 0.001, 0.020 | 0.025 | 0.0314 |
| Great toe extensor strength - left (lbs) | 0.011 | -0.000, 0.022 | 0.051 | 0.0239 |
| Ankle range of motion - left (deg) | 0.000 | -0.002, 0.002 | 0.782 | 0.0005 |
| Knee range of motion - left (deg) | 0.001 | 0.000, 0.002 | 0.007 | 0.0452 |
| Timed up and go (seconds) | -0.015 | -0.021, -0.009 | <0.001 | 0.1271 |
| Five-times-sit-to-stand (seconds) | -0.012 | -0.016, -0.008 | <0.001 | 0.1766 |
| Functional reach (cm) | 0.005 | 0.002, 0.009 | <0.001 | 0.0673 |
| Body sway velocity (mm/s) | -0.037 | -0.061, -0.014 | 0.002 | 0.0597 |
| ABC score | 0.005 | 0.004, 0.006 | <0.001 | 0.3332 |
| Age (years) | -0.003 | -0.006, 0.001 | 0.099 | 0.0171 |
| HbA1c (mmol/mol) | 0.000 | -0.001, 0.001 | 0.875 | 0.0002 |
| Diabetes duration (years) | -0.003 | -0.006, -0.001 | 0.004 | 0.0514 |
| BMI (kg/m^2^) | -0.007 | -0.011, -0.002 | 0.004 | 0.0509 |
| Burning pain | -0.126 | -0.174, -0.078 | <0.001 | 0.1436 |
| Gender, female | -0.104 | -0.151, -0.057 | <0.001 | 0.1074 |
| Ethnicity, others | 0.057 | 0.000, 0.113 | 0.049 | 0.0242 |
| Ever smoker | 0.071 | 0.005, 0.136 | 0.036 | 0.0277 |
| Ever drinker | 0.071 | 0.001, 0.140 | 0.046 | 0.0250 |
| Secondary school education and above | 0.057 | 0.006, 0.108 | 0.029 | 0.0298 |
| Currently married | 0.016 | -0.037, 0.070 | 0.550 | 0.0023 |
| Unemployed | -0.094 | -0.141, -0.047 | <0.001 | 0.0903 |
| Monthly household income above 2,000 SGD | 0.093 | 0.040, 0.147 | 0.001 | 0.0707 |
| Housing categories |  |  |  |  |
| 3-room | 0.027 | -0.050, 0.105 | 0.273 | 0.0246 |
| 4-room | 0.048 | -0.026, 0.122 |  |  |
| 5-room or above | 0.069 | -0.004, 0.142 |  |  |
| Self-reported history of comorbidities |  |  |  |  |
| High blood pressure | -0.045 | -0.096, 0.006 | 0.085 | 0.0186 |
| High cholesterol/lipids | -0.032 | -0.084, 0.020 | 0.226 | 0.0093 |
| Heart disease | -0.018 | -0.077, 0.040 | 0.535 | 0.0024 |
| Peripheral arterial disease | -0.103 | -0.209, 0.002 | 0.055 | 0.0231 |
| Arthritis | -0.094 | -0.169, -0.020 | 0.013 | 0.0381 |

* From simple linear regressions with EQ5D utility score as dependent variable and listed variables as predictor variables.

**Supplementary Table 3. Correlation matrix of functional status variables**

|  | Dorsiflexion strength - right | Great toe extensor strength - right | Dorsiflexion range of motion - right | Knee range of motion - right | Dorsiflexion strength - left | Great toe extensor strength - left | Dorsiflexion range of motion - left | Knee range of motion - left | Timed up and go | Five-times-sit-to-stand | Functional reach | Body sway velocity |
| --- | --- | --- | --- | --- | --- | --- | --- | --- | --- | --- | --- | --- |
| Ankle dorsiflexion strength - right | 1.0000 |  |  |  |  |  |  |  |  |  |  |  |
| Great toe extensor strength - right | 0.4948 | 1.0000 |  |  |  |  |  |  |  |  |  |  |
| Ankle range of motion - right | 0.1678 | -0.1146 | 1.0000 |  |  |  |  |  |  |  |  |  |
| Knee range of motion - right | 0.1110 | 0.2272 | -0.2045 | 1.0000 |  |  |  |  |  |  |  |  |
| Ankle dorsiflexion strength - left | 0.8676 | 0.3981 | 0.2243 | 0.0993 | 1.0000 |  |  |  |  |  |  |  |
| Great toe extensor strength - left | 0.4428 | 0.8857 | -0.0401 | 0.2226 | 0.4072 | 1.0000 |  |  |  |  |  |  |
| Ankle range of motion - left | 0.1191 | -0.0591 | 0.6782 | -0.2250 | 0.1371 | -0.0321 | 1.0000 |  |  |  |  |  |
| Knee range of motion - left | 0.0670 | 0.1542 | -0.1930 | 0.9551 | 0.0729 | 0.1556 | -0.2274 | 1.0000 |  |  |  |  |
| Timed up and go | -0.2882 | -0.3248 | -0.0683 | -0.2884 | -0.2701 | -0.3737 | -0.0484 | -0.2762 | 1.0000 |  |  |  |
| Five-times-sit-to-stand | -0.2876 | -0.2130 | -0.1269 | -0.0939 | -0.2905 | -0.2001 | -0.0518 | -0.0742 | 0.5122 | 1.0000 |  |  |
| Functional reach | 0.1288 | 0.2042 | -0.0096 | 0.2392 | 0.0925 | 0.2728 | -0.0807 | 0.2474 | -0.3158 | -0.2616 | 1.0000 |  |
| Body sway velocity | 0.0476 | 0.0077 | -0.0984 | 0.0349 | 0.0244 | 0.0232 | -0.0999 | 0.0019 | 0.1859 | 0.1608 | -0.1611 | 1.0000 |

**Supplementary Table 4. Summary of results from SEM analysis**

| **Parameters** | **Estimate** | **SE** | **p-value** |
| --- | --- | --- | --- |
| Direct effects |  |  |  |
| DPN 🡪 EQ5D value | 0.058 | 0.020 | 0.004 |
| DPN 🡪 FTSTS | -1.868 | 0.878 | 0.033 |
| DPN 🡪 body sway velocity | -0.498 | 0.157 | 0.001 |
| DPN 🡪 ABC score | 5.798 | 2.606 | 0.026 |
| FTSTS 🡪 ABC score | -1.815 | 0.231 | <0.001 |
| ABC score 🡪 EQ5D value | 0.004 | 0.001 | <0.001 |
| Body sway velocity 🡪 EQ5D value | -0.014 | 0.010 | 0.167 |
| Disturbance variances |  |  |  |
| EQ5D value | 0.015 | 0.002 |  |
| Sit stand 5 times | 30.816 | 3.445 |  |
| Body sway velocity | 0.985 | 0.110 |  |
| ABC score | 264.152 | 29.533 |  |
|  |  |  |  |

**Supplementary Table 5. Bivariate associations with EQ5D utility scores (among DPN group only)**

| **Variables** | **Estimate** | **95% CI** | **p-value** | **R^2^** |
| --- | --- | --- | --- | --- |
| Ankle dorsiflexion strength - right (lbs) | 0.004 | -0.008, 0.017 | 0.512 | 0.0055 |
| Great toe extensor strength - right (lbs) | -0.007 | -0.024, 0.011 | 0.440 | 0.0077 |
| Ankle range of motion - right (deg) | 0.001 | -0.002, 0.004 | 0.470 | 0.0067 |
| Knee range of motion - right (deg) | 0.002 | 0.000, 0.003 | 0.029 | 0.0595 |
| Ankle dorsiflexion strength - left (lbs) | 0.004 | -0.009, 0.017 | 0.582 | 0.0039 |
| Great toe extensor strength - left (lbs) | 0.004 | -0.013, 0.020 | 0.644 | 0.0027 |
| Ankle range of motion - left (deg) | -0.001 | -0.003, 0.001 | 0.386 | 0.0097 |
| Knee range of motion - left (deg) | 0.001 | -0.000, 0.003 | 0.074 | 0.0402 |
| Timed up and go (seconds) | -0.008 | -0.014, -0.001 | 0.018 | 0.0693 |
| Five-times-sit-to-stand (seconds) | -0.007 | -0.012, -0.002 | 0.008 | 0.0870 |
| Functional reach (cm) | 0.004 | -0.000, 0.009 | 0.059 | 0.0451 |
| Body sway velocity (mm/s) | -0.017 | -0.040, 0.007 | 0.157 | 0.0255 |
| ABC score | 0.003 | 0.002, 0.004 | <0.001 | 0.2203 |
| Age (years) | 0.003 | -0.002, 0.008 | 0.211 | 0.0200 |
| HbA1c (mmol/mol) | -0.008 | -0.025, 0.009 | 0.335 | 0.0121 |
| Diabetes duration (years) | -0.002 | -0.005, 0.001 | 0.129 | 0.0292 |
| Burning pain | -0.084 | -0.143, -0.025 | 0.006 | 0.0945 |
| BMI (kg/m^2^) | -0.006 | -0.011, 0.000 | 0.052 | 0.0475 |
| Gender, female | -0.108 | -0.165, -0.051 | <0.001 | 0.1531 |
| Ethnicity, others | 0.073 | -0.006, 0.152 | 0.071 | 0.0412 |
| Ever smoker | 0.079 | 0.003, 0.156 | 0.043 | 0.0515 |
| Ever drinker | 0.120 | 0.035, 0.205 | 0.006 | 0.0923 |
| Secondary school education and above | 0.023 | -0.041, 0.087 | 0.474 | 0.0066 |
| Currently married | 0.021 | -0.044, 0.086 | 0.518 | 0.0054 |
| Unemployed | -0.027 | -0.091, 0.036 | 0.395 | 0.0093 |
| Monthly household income above 2,000 SGD | 0.000 | -0.001, 0.001 | 0.966 | 0.0000 |
| Housing categories |  |  |  |  |
| 3-room | -0.013 | -0.105, 0.080 | 0.327 | 0.0441 |
| 4-room | -0.037 | -0.122, 0.049 |  |  |
| 5-room or above | 0.038 | -0.046, 0.123 |  |  |
| Self-reported history of comorbidities |  |  |  |  |
| High blood pressure | -0.093 | -0.156, -0.030 | 0.004 | 0.0992 |
| High cholesterol/lipids | -0.025 | -0.091, 0.041 | 0.448 | 0.0074 |
| Heart disease | -0.012 | -0.082, 0.058 | 0.730 | 0.0015 |
| Peripheral arterial disease | -0.063 | -0.171, 0.045 | 0.249 | 0.0170 |
| Arthritis | -0.108 | -0.214, -0.002 | 0.045 | 0.0504 |

**Supplementary Table 6. Independent factors associated with HRQoL (among DPN group only)**

| **Model** | **Variables** | **Estimate^a^** | **95% CI** | | **p** | **Adj. R^2^** |
| --- | --- | --- | --- | --- | --- | --- |
| Model 1^b^ | Sit stand 5 times | -0.004 | -0.009, 0.000 | 0.066 | | 0.3428 |
|  | Gender | -0.057 | -0.112, -0.003 | 0.040 | |  |
|  | History of hypertension | -0.100 | -0.157, -0.044 | 0.001 | |  |
|  | Drinking status | 0.097 | 0.018, 0.176 | 0.017 | |  |
|  | Burning pain | -0.062 | -0.115, -0.009 | 0.023 | |  |
| Model 2^c^ | Sit stand 5 times | -0.002 | -0.007, 0.004 | 0.539 | | 0.3664 |
|  | ABC score | 0.001 | 0.000, 0.003 | 0.057 | |  |
|  | Gender | -0.054 | -0.108, -0.000 | 0.050 | |  |
|  | History of hypertension | -0.087 | -0.145, -0.030 | 0.003 | |  |
|  | Drinking status | 0.078 | -0.003, 0.158 | 0.058 | |  |
|  | Burning pain | -0.053 | -0.105, -0.000 | 0.050 | |  |

^a^Beta coefficient from multivariable linear regression

^b^Variables considered: knee ROM, TUG, FTSTS, functional reach, BMI, gender, ethnicity, smoking status, drinking status, history of hypertension, burning pain

^c^Variables considered: knee ROM, TUG, FTSTS, functional reach, BMI, gender, ethnicity, smoking status, drinking status, history of hypertension, ABC score, burning pain
